# Does tumor size have prognostic value in patients undergoing lymphadenectomy in endometrioid-type endometrial cancer confined to the uterine corpus?

**DOI:** 10.3906/sag-1902-224

**Published:** 2019-10-24

**Authors:** Caner ÇAKIR, İsmet Çigdem KILIÇ, Dilek YÜKSEL, Yalın Ay KARYAL, Işın ÜREYEN, Gökhan BOYRAZ, Yasin DURMUŞ, Murat GÜLTEKİN, Nejat ÖZGÜL, Mustafa Alper KARALÖK, Mehmet Coşkun SALMAN, Kunter YÜCE, Ahmet Taner TURAN

**Affiliations:** 1 Gynecologic Oncology Clinic, Etlik Zübeyde Hanım Women’s Health Teaching and Research Hospital,University of Health Sciences, Ankara Turkey; 2 Gynecologic Oncology Clinic, Antalya Training and Research Hospital, University of Health Sciences, Antalya Turkey; 3 Gynecologic Oncology Clinic, Faculty of Medicine, Hacettepe University, Ankara Turkey

**Keywords:** Tumor size, lymphadenectomy, endometrial cancer

## Abstract

**Background/aim:**

We aimed to define the effect of tumor size on recurrence and survival rates in patients with stage I–II endometrioid-type endometrial cancer.

**Materials and methods:**

A total of 550 patients who had total abdominal hysterectomy, bilateral salpingo-oophorectomy, and pelvic-paraaortic lymphadenectomy were included. Patients with extrauterine spread, sarcomatous components, or synchronized tumor and those who did not undergo lymphadenectomy or did not have data on tumor size were excluded.

**Results:**

The median tumor size was 35 mm (range: 3–335 mm). According to the 2009 International Federation of Obstetrics and Gynecology (FIGO) criteria, 245 cases were defined as stage IA, 271 as stage IB, and 34 as stage II. The 5-year disease-free survival (DFS) rate was 92% and the 5-year disease-specific survival (DSS) rate was 99%. The effects of prognostic factors on DFS were evaluated. Older age, stage II disease, deep myometrial invasion, and receiving adjuvant radiotherapy were associated with decreased DFS. There was no statistically significant association between tumor size and DFS. The 5-year DFS for patients with a tumor diameter of <35 mm, which was the median tumor size of the entire group, was 94%, while it was 89% for patients having a tumor diameter of >35 mm (P = 0.128).

**Conclusion:**

Tumor size was not a risk factor predicting recurrence in patients with stage I or II endometrioid-type endometrial cancer who had lymphadenectomy.

## 1. Introduction

Endometrial cancer (EC) is the most common malignancy of the female genital tract in developed countries, with 80% of newly diagnosed patients having endometrioid-type tumors [1,2]. The tumor is limited to the uterine corpus in 70% of patients at the time of initial diagnosis, and patients with early-stage and low-grade tumors have a 5-year survival rate over 90% [3,4]. Age, cancer stage, cell type, grade, depth of myometrial invasion, the presence of cervical invasion, and presence of nodal/nonnodal extrauterine disease are the major clinical and surgical factors determining recurrence and survival rates in EC [5,6].

Tumor size, which is another prognostic factor, is associated with poor surgicopathological factors, primarily with lymphatic spread [7–9]. However, the relationship between tumor size and recurrence and survival rates is not clear. Todo et al. showed that tumor volume was an independent prognostic factor for poor survival rates in their study, wherein the patients were divided into groups according to tumor volume. In multivariate analysis, patients who had a volume index of ≥36 cm³ were found to have a mortality rate twice that of other patients (HR = 1.98, 95% CI: 1.25–3.3; P = 0.0036) [10]. This result was supported by other studies [11–13]. On the other hand, a study by Shah et al., in which 345 patients with endometrial cancer of all tumor types were evaluated and about 85% of them underwent surgical staging, did not show tumor size to be predictive of recurrence [14]. Similarly, Ozgul et al. showed that tumor size did not correlate with survival in 250 surgically staged patients who had stage 2 endometrial cancers [15]. Moreover, tumor size and local surgicopathological factors are not associated with extrauterine dissemination in patients with high-risk tumor types. Extrauterine disease may be observed in patients with serous uterine cancers without myometrial invasion regardless of tumor size [16,17].

Thus, the relationship between tumor size and prognosis is unclear. The question of whether this relationship is the result of the association of tumor size with poor surgical prognostic factors, especially with lymphatic metastases, or whether it is the direct effect of tumor size on prognosis still remains. On the other hand, patients with high-risk tumor types have high rates of extrauterine spread regardless of tumor size and the presence of myometrial invasion. Therefore, the present study included patients with endometrioid-type endometrial cancer without lymphatic dissemination and without extrauterine nonnodal spread, as confirmed in the final pathological examination, and evaluated the possible effect of tumor size on prognosis. For this purpose, we aimed to investigate the effect of tumor size on recurrence and survival rates in patients with stage I and II endometrioid-type endometrial cancer who underwent lymphadenectomy.

## 2. Materials and methods

### 2.1. Study population

A total of 550 patients were enrolled in the study group. Of this group, 280 patients had undergone total abdominal hysterectomy, bilateral salpingo-oophorectomy, and lymphadenectomy at the University of Health Sciences Etlik Zübeyde Hanım Gynecologic Oncology Clinic between January 1993 and May 2013. The remaining 270 patients had undergone total abdominal hysterectomy, bilateral salpingo-oophorectomy, and lymphadenectomy between November 1996 and June 2014 at the Hacettepe University Faculty of Medicine’s Division of Gynecologic Oncology. Patients were excluded from the study when they were diagnosed to have nodal/nonnodal extrauterine spread, nonendometrioid-type tumors, synchronized tumors, or sarcomatous components of the tumor in the final pathological examination. Patients who received neoadjuvant therapy, who did not undergo lymphadenectomy, or who had missing data on tumor size were also excluded. Patient data were obtained from electronic databases, patient files, and pathology reports. Patients were staged according to the 2009 criteria described by the International Federation of Obstetrics and Gynecology (FIGO). Tumor size was obtained from pathology reports. The largest diameter was accepted as the tumor size. Institutional review board approval was obtained from the institution’s local ethical committee (Approval Code: 2018/228). 

### 2.2. Treatment and follow-up

Frozen section examination was routinely used in the management of endometrial cancers in both clinics. Staging surgery was performed in all patients, except for those with grade 1 and 2 endometrioid-type tumors, less than 1/2 myometrial invasion, or a tumor size smaller than 2 cm. The staging surgery was standardized as total abdominal hysterectomy + bilateral salpingo-oophorectomy + systematic pelvic and paraaortic lymphadenectomy + cytological sampling + omentectomy or omental biopsy. Lymphadenectomy was performed in the majority of patients by skeletonizing the vessels in the pelvic and paraaortic region. However, paraaortic lymphadenectomy was not included in the surgical procedure in a small group of the patients at the discretion of the surgeon (n = 20).

The necessity and use of adjuvant chemotherapy and radiotherapy (only radiotherapy or concomitant chemoradiotherapy), and the type of radiotherapy (vaginal brachytherapy [VBT] or external beam radiotherapy [EBRT] plus VBT), were decided by the gynecological oncology tumor boards in both clinics. The recurrence of the disease within 1 month after the initial surgical treatment or, when adjuvant therapy was applied, the progression of the disease during adjuvant therapy was accepted as refractory disease. After no signs of the disease were present in the follow-up visit 1 month after the adjuvant therapy and during the follow-up period, an occurrence of treatment failure was defined as recurrence. Recurrences were accepted as pelvic recurrences when they occurred in the vaginal wall and/or in the pelvic sidewall below the pelvic brim, or as upper-abdominal recurrences when they occurred between the pelvic brim and the diaphragm. All other recurrences were accepted as extraabdominal recurrences. Ascites and peritonitis carcinomatosa were accepted as upper-abdominal recurrences, and recurrences in the liver parenchyma, bone, and skin were accepted as extraabdominal recurrences. Recurrences were defined by clinical, radiological, and histological findings obtained from pelvic and systemic examinations, abdominal X-rays, abdominopelvic- and thoracic-computed tomography, or magnetic resonance imaging. The decision for recurrence treatment was made by the gynecological oncology tumor boards in both clinics.

After the surgery or adjuvant therapy, if applied, patients were followed every 3 months for 2 years, every 6 months until the fifth year, and yearly thereafter. During the follow-up period, physical examinations of the pelvis, abdominopelvic imaging with ultrasound, and complete blood count and blood biochemical tests were performed. A chest X-ray was utilized yearly or in the case of clinical suspicion. Thoracic and/or abdominal computerized tomography was used when needed. CA 125 level was utilized in the follow-up period, even though it was not used routinely. 

### 2.3. Statistical analysis

The time from the surgery to the recurrence or to the last visit was called disease-free survival (DFS). The time from the surgery to death due to endometrial cancer or to the last visit was defined as disease-specific survival (DSS). The time from the surgery to the recurrence was defined as time to recurrence (TTR). The Kaplan–Meier method was used for performing the survival analyses. A log-rank test was utilized to determine whether categorical variables had statistically significant effects on DFS and DSS. Factors with a P-value of <0.25 were included in the multivariate analysis. The statistical analyses were performed using SPSS 11.5 for Windows. The cut-off for statistical significance was set at P < 0.05.

## 3. Results

A total of 550 patients with stage I and II endometrioid-type endometrial cancers were included in the study. The mean age was 58.5 years (range: 33–92 years). Only pelvic lymphadenectomy without paraaortic lymphadenectomy was performed in 20 patients (3.6%). The median number of lymph nodes removed was 39 (range: 1–122), and more than 20 lymph nodes were removed in 80% of the patients. The median tumor size was 35 mm (range: 3–335 mm). In the study group, the tumor size was 20 mm or smaller in 129 patients (23.5%), 21–30 mm in 127 patients (23.1%), 31-40 mm in 129 patients (23.5%), 41–50 mm in 72 patients (13.1%), and 51 mm and over in 93 patients (16.9%). According to the FIGO 2009 staging system, 245 patients (44.5%) were diagnosed with a stage IA disease, 271 patients (49.3%) with a stage IB disease, and 34 patients (6.2%) with a stage II disease. According to the FIGO grading system, 289 patients had a grade 1 disease, 182 had grade 2, and 74 had grade 3. The myometrial invasion was ≥1/2 in 227 patients, while 55 patients did not have myometrial invasion. A lymphovascular invasion was detected in 103 patients, and a cervical glandular ±stromal involvement was observed in 69 patients. Peritoneal cytology was positive only in 1 patient (Table 1).

**Table 1:** Patients’ characteristics.

Characteristics		Entire cohort (n = 550)		Recurrent patients (n = 25)	
		n / Mean	% / Median (range)	n / Mean	% / Median (range)
Age at initial diagnosis		58.5	58 (33–92)	61	61 (37–77)
Disease-free interval (month)		20.2	17 (5–46)	-	-
Number of removed lymph nodes		43.3	39 (1–122)	46	38 (9–118)
Tumor size at initial diagnosis (mm)		37.4	35 (3–335)	42	40 (15–100)
Tumor size atinitial diagnosis (mm)	≤20	23.5	129	3	12	21–30	23.1	127	5	20	31–40	23.5	129	8	32	41–50	13.1	72	5	20	≥51	16.9	93	4	16
	FIGO 2009 stage	IA	44.5	245	11	44	IB	49.3	271	11	44	II	6.2	34	3	12
		FIGO grade	1	52.5	289	7	28	2	33.1	182	14	56	3	13.5	74	4	16	Not reported	0.9	5	-	-
			Depth of myometrialinvasion	No invasion	10.0	55	-	-	<1/2	48.7	268	5	20	≥1/2*	41.3	227	20	80
	Lymphovascular space invasion			Negative	58.7	323	16	64	Positive	18.7	103	4	16	Not reported	22.5	124	5	20
Cervical invasion				Negative	87.5	481	20	80	Glandular	6.4	35	2	8	Stromal	6.2	34	3	12	Not reported	-	-	-	-
		Peritoneal cytology	Negative	96.4	530	21	80	Positive	0.2	1	0	0	Not reported	3.4	19	4	16
	Adjuvant therapy		Not received	66	363	11	44	Received	34	187	14	56
			Type of adjuvant therapy	Radiotherapy	31.8	175	13	52	Concomitant chemoradiotherapy	0.7	4	1	4	Chemotherapy	0.7	4	-	-	Sandwich therapy †	0.5	3	-	-	Chemotherapy followed by radiotherapy	0.2	1	-	-
Recurrence	Negative	95.5		525			Positive	4.5	25		
	Site of recurrence	Only pelvic		2.7	15			Only upper abdominal	0.5	3			Only extra abdominal	1.1	6			Pelvic + upper abdominal	-	-			Pelvic + extra abdominal	-	-			Upper abdominal + extra abdominal	0.2	1			Pelvic + upper abdominal + extra abdominal	-	-		

* Except for uterine serosal invasion† Three cycles of paclitaxel and carboplatin followed by radiotherapy followed by 3 cycles of paclitaxel and carboplatin.

Of the study patients, 187 (34%) received adjuvant treatments. Among these patients, 175 patients received only radiotherapy, 4 received concomitant chemotherapy and radiotherapy, 4 received chemotherapy alone, 3 received sandwich therapy (initial 3 cycles of paclitaxel and carboplatin followed by radiotherapy, followed by 3 cycles of paclitaxel and carboplatin), and 1 received chemotherapy followed by radiotherapy (Table 1).

The median follow-up period was 29 months (range: 1–167 months). During this period, 25 patients (4.5%) had recurrences and 2 patients (0.4%) died of the disease. Median TTR was 17 months (range: 5–46 months). Recurrences were present only in the pelvic region in 15 patients. Extraabdominal recurrences were present in 7 patients. Recurrent disease was defined by clinical, laboratory, and imaging methods. Clinical, surgical, and pathological features of the entire cohort and of patients with recurrence are presented in detail in Table 1.

The 5-year DFS and the 5-year DSS in the entire cohort were 92% and 99%, respectively. The effects of the prognostic factors on DFS were evaluated since only 2 patients died during the follow-up period. Older age, stage II disease, deep myometrial invasion, and receiving adjuvant radiotherapy were associated with decreased DFS (Table 2). However, poorer surgical and pathological factors were present in the group receiving adjuvant radiotherapy. This group of patients had a higher disease stage (P < 0.001), deeper myometrial invasion (P < 0.001), and larger lymphovascular space invasion (P < 0.001); furthermore, a higher number of patients in this group had grade 3 disease (P < 0.001). Glandular and/or stromal cervical invasion was more frequent (P < 0.001) and the tumor size was larger in this group (P = 0.025). Moreover, the group receiving adjuvant radiotherapy tended to be older (P = 0.075). However, the numbers of excised lymph nodes were similar between the two groups (P = 0.106).

**Table 2:** Univariate analysis of the factors predicting disease-free survival.

Factors		5-year disease-free survival (%)	P-value
Age at initial diagnosis*	<58 years	96	0.005	≥58 years	88
	Number of lymph nodes*	≤38		92	0.841	≥39	93
2009 FIGO stage		I	94	0.024		II	88
	Tumor size*	≤35 mm	95		0.128	≥36 mm	89
Tumor size		≤20 mm	97	0.398		21–30 mm	94	31–40 mm	88	41–50 mm	88	≥51 mm	93
	FIGO grade	1 and 2	92		0.916	3	92
		Depth of myometrial invasion	<1/2			97	<0.001	≥1/2 †	86
	Lymphovascular space invasion		Negative		91	0.539		Positive	95
		Cervical invasion ‡	Negative		93		0.341	Positive	88
Adjuvant therapy §	Not received		94	0.058	Received	90	
	Adjuvant radiotherapy	Not received	94		0.039	Received	89

* Median value.† Except for uterine serosal invasion.‡ Glandular ±stromal invasion.§ Radiotherapy and/or chemotherapy.

There was not a statistically significant association between tumor size and DFS. The 5-year DFS for patients with tumor diameter equal to or less than 35 mm, which was the median tumor size of the entire group, was 94%, and it was 89% for patients with a tumor diameter greater than 35 mm (P = 0.128) (Figure). When the patients were divided into the groups according to tumor diameter as ≤20 mm, 21–30 mm, 31–40 mm, 41–50 mm, and ≥51 mm, no statistically significant differences were detected between the groups regarding DFS (Table 2).

**Figure:**
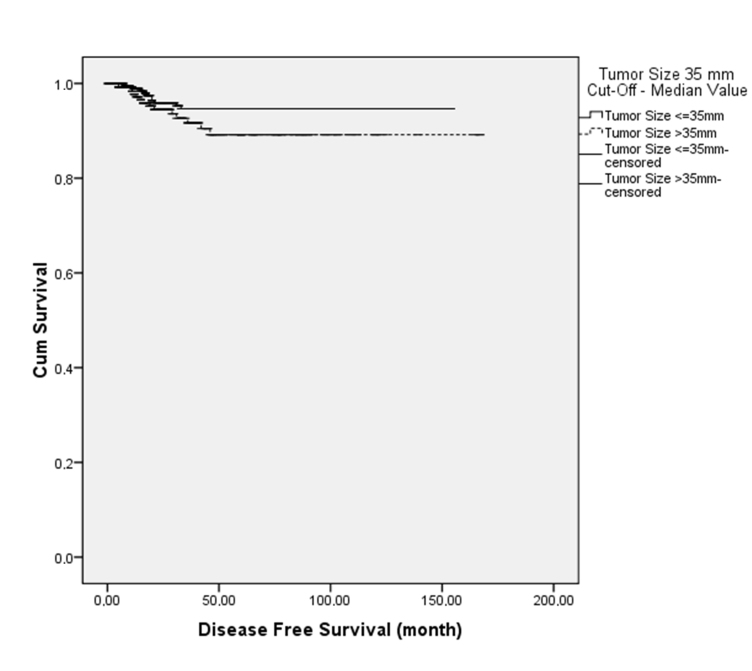
The 5-year DFS for patients by tumor diameter.

The factors detected to have a P-value under 0.25 in the univariate analysis were included in multivariate analysis. These factors included age (≥58 years vs. <58 years), stage (FIGO stage I vs*.* II) (P < 0.05), tumor size (>35 mm vs. ≤35 mm), depth of myometrial invasion (<1/2 vs. ≥1/2), and adjuvant radiotherapy (received vs. not received). After the correlation between these factors was determined, a model was constructed for multivariate analysis using age, tumor size, and adjuvant radiotherapy. However, no independent prognostic factors for DFS could be identified (Table 3).

**Table 3:** Results of the multivariate analyses of the odds ratios in the exact logistic regression model with recurrence as the dependent variable.

Variables	Odds ratio	95% Confidence interval	P-value
Age* (≥58 years vs. <58 years)	1.113	0.423–2.928	0.829
Tumor size* (>35 mm vs. ≤35 mm)	1.274	0.526–3.083	0.591
Adjuvant RT (received vs. not received)	1.581	0.626–3.992	0.332

* Median value.

## 4. Discussion

The prognostic effect of the tumor size in EC is controversial. It has not been clarified yet whether there is an association between tumor size and poor prognostic factors, or whether poor prognosis occurs solely due to the prognostic value of the tumor size itself. There are additional factors complicating assessment of the prognostic effect of the tumor size, such as the multifocal presence of the tumor in the endometrial cavity, variable localization of tumors, and the irregular surface of the tumor, which complicates measuring the tumor size.

The relationship between tumor size and poor prognostic factors, especially lymphatic spread, has been identified. Lymphatic metastases are considered to increase as tumor size increases [7–13,16,17]. Vargas et al. evaluated the Surveillance, Epidemiology, and End Results (SEER) data in their study, wherein patients were divided into risk groups based on the presence of lymphatic spread. In that study, lymphatic involvement was 1.4% in the patients in the low-risk group who had a tumor size of <2 cm, a grade 1–2 tumor diagnosed histologically, and myometrial invasion of <50%. The same study reported that lymphatic involvement was 6.4% in patients in the high-risk group (P < 0.001). Moreover, an increase in tumor size was found to increase lymph node metastases in the multivariate logistic regression analysis in their study (OR = 1.07, 95% CI: 1.05–1.09; P < 0.005) [18]. Boyraz et al. showed that tumor size was associated with lymphatic metastases in their study including 191 stage 1A EC cases. Lymphatic spread was detected in 6.28% of the patients with a tumor size of ≥2 cm, and no lymphatic metastases were observed in the patients with a tumor size of <2 cm (P = 0.009) [19]. Shah et al. showed that the association between tumor size and the presence of nodal metastasis was not statistically significant in the multivariate analysis in their study (OR = 1.3, 95% CI: 1.0–1.8; P > 0.05) [20].

Shinck et al. showed that tumor size greater than 2 cm was associated with nodal metastases and poor survival in patients having less than 50% myometrial invasion in grade 1 and 2 and stage I endometrial carcinoma [8]. Sozzi et al. found that an increase in tumor size was a poor prognostic factor for recurrence in their study, wherein all tumor types were included and patients were assigned to the study groups based on the European Society for Medical Oncology (ESMO), European Society for Radiotherapy & Oncology (ESTRO), and European Society of Gynaecological Oncology (ESGO) criteria (n = 1166). The authors reported that recurrence-free survival was significantly lower in patients with tumor size greater than 25 mm (P < 0.0001) [21]. Senol et al. supported this result with their study (n = 152), in which they included all histological types of tumors. They found that tumor size was associated with DFS (OR = 1.2, 95% CI: 1.016–1.394; P = 0.031). In univariate analysis, the recurrence rate was 21.9% in the group with tumors larger than 3.75 cm, whereas it was 3.4% in patients with tumors smaller than 3.75 cm (OR = 7.9, 95% CI: 2.2–28.9, P < 0.001) [22]. However, the relationship between tumor size and oncological outcome in that study could be explained by the fact that a lymphadenectomy surgery was not performed and the exact stage of the disease could not be defined. Chattopadhyay et al. reported that an increase in tumor size affected the rates of recurrence and death in patients who did not undergo lymphadenectomy [23]. On the other hand, Shah et al. evaluated 345 patients and included all tumor types in their study, in which 85% of the patients underwent surgical staging. They determined that tumor size was not an independent prognostic factor for recurrence [20]. Similarly, a retrospective analysis of 250 surgically staged cases by Ozgul et al. showed that 5-year DFS and OS did not display differences with increased tumor size [15]. Moreover, an ESMO-ESGO-ESTRO study stated that tumor size should not be used as a risk factor in the risk classification of EC [24]. Tumor size was not a prognostic factor for DFS in our study, which included 550 patients with stage I and II endometrioid-type endometrial cancer, and in which nodal spread was determined by lymphadenectomy.

The retrospective nature was the most important limitation of the present study. Another limitation was related to the nature of the tumoral structure, along with the number of tumoral foci and the localization of the tumor. However, our strict exclusion criteria allowed us to create a homogeneous cohort. The strength of the study was that the confinement of cancer in the uterine corpus was proven by a lymphadenectomy surgery. The previously defined association between tumor size and nodal spread was eliminated by including the patients who had not undergone lymphadenectomy. The median number of removed lymph nodes was 39 in the study group, and more than 20 lymph nodes were removed in 80% of the patients. Moreover, the exclusion of tumors directly based on the presence of an extrauterine spread provided a clear assessment of the relationship between tumor size and survival. Additionally, the study cohort consisted of a large number of patients.

In conclusion, tumor size was not a risk factor for recurrence in patients with stage I and II endometrioid-type endometrial cancer who underwent lymphadenectomy surgery. Therefore, tumor size should not be taken into consideration while planning the treatment protocol in this group.

## References

[ref1] (1991). The epidemiology of endometrial cancer. Gynecologic Oncology.

[ref3] (2007). Endometrial carcinoma: Pathhology and genetics. Pathology.

[ref6] (2006). Carcinoma of the corpus uteri. FIGO Annual Report.

[ref10] (2013). Endometrial cancer: ESMO clinical practice guidelines for diagnosis, treatment and follow-up. Annals of Oncology.

[ref13] (2009). Revised FIGO staging for carcinoma of the vulva, cervix, and endometrium. International Journal of Gynecology &amp; Obstetrics.

[ref15] (1987). Tumor size in endometrial cancer: a prognostic factor for lymph node metastasis. Obstetrics and Gynecology.

[ref17] (2013). Preoperative biopsy and intraoperative tumor diameter predict lymph node dissemination in endometrial cancer. Gynecologic Oncology.

[ref26] (2013). Association between tumor diameter and lymphovascular space invasion among women with early-stage endometrial cancer. International Journal of Gynecology &amp; Obstetrics.

[ref28] (2013). Tumor volume successively reflects the state of disease progression in endometrial cancer. Gynecologic Oncology.

[ref37] (1994). Karnovsky memorial lecture. Natural history of small breast cancers. Journal Clinical of Oncology.

[ref40] (2000). Potential therapeutic role of para-aortic lymphadenectomy in node-positive endometrial cancer. Gynecologic Oncology.

[ref43] (1960). Tovell HM. American Journal of Obstetrics and Gynecology.

[ref46] (2005). Does size matter? Tumor size and morphology as predictors of nodal status and recurrence in endometrial cancer. Gynecologic Oncology.

[ref53] (2018). Oncological outcomes of stage II endometrial cancer: a retrospective analysis of 250 cases. International Journal of Gynecological Cancer.

[ref58] (1987). Uterine papillary serous carcinoma. Obstetrics and Gynecology.

[ref60] (1995). Early pathologic stage clear cell carcinoma and uterine papillary serous carcinoma of the endometrium: comparison of clinicopathologic features and survival. International Journal of Gynecological Pathology.

[ref62] (2014). Tumor size, depth of invasion, and histologic grade as prognostic factors of lymph node involvement in endometrial cancer: a SEER analysis. Gynecologic Oncology.

[ref68] (2017). Incidence of lymph node metastasis in surgically staged FIGO IA G1/G2 endometrial cancer with a tumor size of more than 2 cm. International Journal of Gynecological Cancer.

[ref70] (1987). Surgical pathologic spread patterns of endometrial cancer. Cancer.

[ref73] (2018). Tumor size, an additional risk factor of local recurrence in low-risk endometrial cancer: a large multicentric retrospective study. International Journal of Gynecological Cancer.

[ref79] (2015). Tumor diameter for prediction of recurrence, disease free and overall survival in endometrial cancer cases. Asian Pacific Journal of Cancer Prevention.

[ref82] (2013). Tumor size: a better independent predictor of distant failure and death than depth of myometrial invasion in International Federation of Gynecology and Obstetrics stage I endometrioid endometrial cancer. International Journal of Gynecological Cancer.

[ref84] (2017). European guidelines (ESMO-ESGO-ESTRO consensus conference) for the management of endometrial cancer. Bulletin du Cancer.

